# The Positive Choices trial: update to study protocol for a phase-III RCT trial of a whole-school social-marketing intervention to promote sexual health and reduce health inequalities

**DOI:** 10.1186/s13063-022-06191-2

**Published:** 2022-04-11

**Authors:** Ruth Ponsford, Rebecca Meiksin, Elizabeth Allen, G. J. Melendez-Torres, Steve Morris, Catherine Mercer, Rona Campbell, Honor Young, Maria Lohan, Karin Coyle, Chris Bonell

**Affiliations:** 1 Faculty of Public Health & Policy, London School of Hygiene and Tropical Medicine, 15-17 Tavistock Place, London, WC1H 9SH UK; 2 Faculty of Epidemiology and Population Health, London School of Hygiene and Tropical Medicine, Keppel Street, London, WC1E 7HT UK; 3 University of Exeter College of Medicine and Health, South Cloisters, St Luke’s Campus, Heavitree Road, Exeter, EX1 2LU UK; 4 Cambridge University Department of Public Health & Primary Care, Strangeways Research Laboratory, Worts Causeway, Cambridge, CB1 8RN UK; 5 UCL Institute for Global Health, 3rd Floor Mortimer Market Centre, off Capper Street, London, WC1E 6JB UK; 6 University of Bristol, 1-5 Whiteladies Road, Clifton, Bristol, BS8 1NU UK; 7 DECIPHer, Cardiff School of Social Sciences, Cardiff University, 1-3 Museum Place, Cardiff, CF10 3BD UK; 8 School of Nursing and Midwifery, Medical Biology Centre, 97 Lisburn Road, Belfast, BT9 7BL UK; 9 ETR Associates, Scotts Valley, USA

**Keywords:** Schools, Adolescents, Sexual health, Sexual competence, Pregnancy, Sexually transmitted infections, Whole-school, Cluster trials, Public health

## Abstract

**Background:**

Positive Choices is a whole-school social-marketing intervention to promote sexual health among secondary school students. Intervention comprises the following: school health promotion council involving staff and students coordinating delivery, student survey to inform local tailoring, teacher-delivered classroom curriculum, student-run campaigns, parent information and review of sexual/reproductive health services to inform improvements. This trial builds on an optimisation/pilot-RCT study which met progression criteria, plus findings from another pilot RCT of the Project Respect school-based intervention to prevent dating and relationship violence which concluded such work should be integrated within Positive Choices. Young people carry a disproportionate burden of adverse sexual health; most do not report competence at first sex. Relationships and sex education in schools can contribute to promoting sexual health but effects are small, inconsistent and not sustained. Such work needs to be supplemented by ‘whole-school’ (e.g. student campaigns, sexual health services) and ‘social marketing’ (harnessing commercial marketing to social ends) approaches for which there is good review-level evidence but not from the UK.

**Methods:**

We will conduct a cluster RCT across 50 schools (minimum 6440, maximum 8500 students) allocated 1:1 to intervention/control assessing outcomes at 33 months. Our primary outcome is non-competent first sex. Secondary outcomes are non-competent last sex, age at sexual debut, non-use of contraception at first and last sex among those reporting heterosexual intercourse, number of sexual partners, dating and relationship violence, sexually transmitted infections and pregnancy and unintended pregnancy for girls and initiation of pregnancy for boys. We will recruit 50 school and undertake baseline surveys by March 2022, implement the intervention over the 2022–2024 school years and conduct the economic and process evaluations by July 2024; undertake follow-up surveys by December 2024; complete analyses, all patient and policy involvement and draft the study report by March 2025 and engage in knowledge exchange from December 2024.

**Discussion:**

This trial is one of a growing number focused on whole-school approaches to public health in schools. The key scientific output will be evidence about the effectiveness, costs and potential scalability and transferability of Positive Choices.

**Trial registration:**

ISRCTN No: ISRCTN16723909. Registered on 3 September 2021.

## Changes to the protocol

### Changes for version 3.0

#### Amendment 1: Option of surveying students online at home

If schools dismiss classes and move to online learning because of COVID infections, we will offer schools the option of surveying students online at home. Schools will send students details of how to log in to surveys using laptops, phones or tablets. Surveys will include links to information boxes explaining key terms informed by the questions students have raised during classroom-based surveys. Students will be asked to skip questions they do not understand or do not wish to answer. Students will be advised to contact their school safeguarding lead for support should they feel confused or upset as a result of completing the questionnaire, with the team briefing safeguarding leads about this and liaising with them to record where this has occurred.

#### Amendment 2: Increase in number of lessons

We will add one additional (core) lesson for year 10 students in intervention group to ensure dating and relationship violence and sexual harassment are adequately addressed [1].

#### Amendment 3: Flexibility for training arrangements

We will provide training to schools more flexibly to fit with school needs. The initial training for school leads in the curriculum will remain as 7 h in total, but this will be delivered in flexible sections to fit school needs. This training will be video-recorded and made available to schools. The training which school leads cascade to other teachers involved in delivering the curriculum will involve a minimum of three hour’s training but organised flexibly to fit school needs.

### Changes for version 4.0: Further amendments to intervention

#### Amendment 4

The intervention has been modified in three ways. First, the initial start-up meeting with schools has been reduced from 2 h to half an hour. Second, the cascading of training by schools internally has been broadened to allow a range of total training duration from 1 to 3 h. Third is additional support in the form of webinars and drop-in sessions. These changes have been made based on advice from the Sex Education Forum as the intervention lead agency. They reflect the need for support to schools to be more flexible to reflect schools’ varying needs.

## Trial status

Schools were recruited September–December 2021, and students are being recruited and surveyed November 2021–March 2022.

## Data Availability

Data will be made available after the main trial analyses have been completed on reasonable request from researchers with ethics approval and a clear protocol.

## Abbreviations

DRV: Dating and relationship violence
RCT: Randomised controlled trial
RIPPLE: Randomised intervention for pupil led sex education in England
